# Fracture en bec de canard de la tuberosité posterieure du calcaneum: à propos d’‘un cas

**DOI:** 10.11604/pamj.2015.20.106.5709

**Published:** 2015-02-05

**Authors:** Ismaïl Hmouri

**Affiliations:** 1 Service de Traumatologie Orthopédie, CHU Avicene, Rabat, Maroc

**Keywords:** Calcaneum, fracture, bec, cannard, Calcaneum, fracture, beak, duck

## Image en medicine

Les fractures de calcaneum constituent 60% des fractures de tarse et 02% de l'ensemle des fractures. La fracture en bec de canard de la tuberosité posterieure, appelée également avulsion de l'insertion du tendon d'achile est une entitée encore plus rare mais de bon pronostic. Dans notre cas, on decrit l'observation d'un jeune homme de 24 ans, sans antecedents pathologiques notables, qui a présenté suite a un accident de sport survenu lors d'un match de foot, après une sensation de craquement au niveau de l'arrière pied gauche, une cheville gauche oedematiée,ecchymotique,avec impotence foctionnelletotale,impossibilité de se tenir debout sur la pointe du pied gauche et un signe de Thompson positif a gauche. Un bilan radiologique fait d'une radiographie standard de la cheville a objective une fracture extra-thalamique du calcaneum interessant la tuberosité posterieure, en bec de canard. La prise en charge a consisté en une intervention chirurgicale sous rachi-anesthesie pendant laquelle on a realisé un vissage direct,completé par une botte platrée en équin pendant 04 semaines, avec interdiction de l'appui, sous couvert d'une anticoagulation a dose prophylactique, suivie après l'ablation du plâtred'une reeducation fonctionnelle. Le résultat avec un recul de 01 an était satisfaisant avec une bonne évolution clinique et radiologique. Les fractures en bec de canard du calcaneum sont rares mais de bon pronostic.

**Figure 1:**
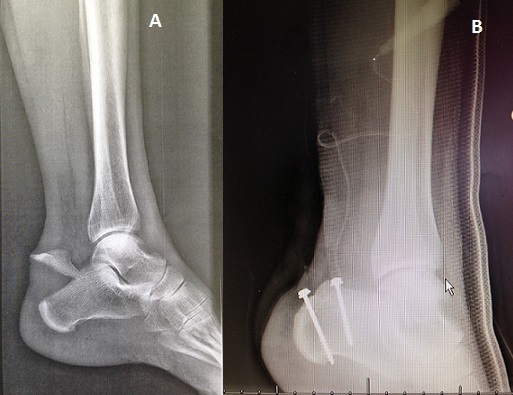
(A) radiologie standard de profile de la cheville gauche objectivant une fracture en bec de canard du calcanéum; (B) radiographie de contrôle de profile montrant l'ostéosynthèse par vissage de la fracture de clacaneum

